# Detecting the Research Trends and Hot Spots in External Irradiation Therapy for Rectal Cancer

**DOI:** 10.7150/jca.69669

**Published:** 2022-04-04

**Authors:** Qian Bi, Zheng Miao, Jing Shen, Hao Wang, Kai Kang, Junjie Du, Fuquan Zhang, Shaoping Fan

**Affiliations:** 1 Department of Radiation Oncology, Peking Union Medical College Hospital, Chinese Academy of Medical Sciences and Peking Union Medical College, Beijing, China.; 2 Department of Oncology, Qingdao Municipal Hospital, School of Medicine, Qingdao University, Qingdao, China.; 3 Department of Radiotherapy, the No 1 People's Hospital, Yangquan, China.; 4 Institute of Medical Information & Library, Chinese Academy of Medical Sciences & Peking Union Medical College, Beijing, China.

**Keywords:** rectal cancer, EBRT, scientometric analysis, global trends, visualization analysis

## Abstract

**Purpose:** The aim of this study is to visualize and analyze the research hot pots in radiation therapy for rectal cancer and provide researchers with a clear and visual reference for subsequent studies.

**Methods:** The literature scientometric analysis about “external beam radiation therapy (EBRT) for rectal cancer” was made through the WoSCC (2010 to 2019). And the data was visualized and analyzed by the Microsoft Office Excel (2019) and CiteSpace (V. 5.7.R1).

**Results:** 4,263 relevant articles were downloaded. The number of published articles per year has been increasing (2010-2020). The United States published the highest number of articles. The UK has the strongest partnerships with other countries/regions. Leiden University has the highest number of published articles and University Texas MD Anderson Cancer Center has extensive collaborations with other academic institutions. The number of articles published in the Diseases of the Colon Rectum is the most, and the Journal of Clinical Oncology is the most cited. 27 articles became the strongest burst citations lasting until 2020. In recent years, a lot of research has been done on “watch and wait”, “short-course radiotherapy”, “MRI”, “neoadjuvant radiotherapy, “MRI in rectal cancer”, “chemotherapy regimen improvement”, “adding adjuvant chemotherapy”, and so on.

**Conclusion:** The current research hot pots may be the “watch and wait”, “short-course radiotherapy”, “MRI”, “neoadjuvant chemoradiotherapy”, “MRI in rectal cancer”, “chemotherapy regimen improvement”, and “adding adjuvant chemotherapy”.

## Introduction

Rectal cancer is the most common cancer of the digestive system and now ranks as the 3^rd^ most commonplace incidence and the 4^th^ major causes of cancer deaths 1. Radiotherapy has a prominent place in the comprehensive treatment plan for rectal cancer patients, including external beam radiation therapy (EBRT) and brachytherapy 2. With advances in radiotherapy technology, EBRT has further increased the target dose while protecting normal tissues, improving disease control rates while reducing the incidence of adverse effects.

CiteSpace is a new software for dosimetric analysis of literature. In other areas, CiteSpace has been widely used to help beginners get started quickly 3, 4. It can overcome the shortcomings of traditional methods and present the structure, mode and distribution of scientific knowledge in a visual way. CiteSpace can scan a vast number of articles to qualitatively and quantitatively evaluate research in nations, institutions, and specific topic areas, making it easier for novices to detect research hotspots graphically 3.

There are constantly new articles summarizing the present situation and progress of EBRT about the area of rectal cancer. However, few studies have been conducted with the help of CiteSpace (V. 5.7.R1) for visualization and analysis of papers. After preliminary literature research, this study is one of the earlier studies that used CiteSpace (V. 5.7.R1) for visual analysis in the field of radiotherapy, which may be more helpful to understand the hot spots and trends of rectal cancer research and promote the in-depth exploration of future studies.

## Materials and methods

### Search strategy and search database

An extensive exploration of published articles in the field of external irradiation for rectal cancer was performed using three phrases (rectal cancer, brachytherapy, and EBRT/radiotherapy) from the Web of Science Core Collection (WoSCC) title 5. Then, two terms (rectal cancer and radiotherapy/EBRT) were combined using Boolean operations (Appendix S1), and then brachytherapy-related articles were excluded, and multiple iterations were performed to make sure the consistency between the searched terms and the results 5.

**Figure 1:**
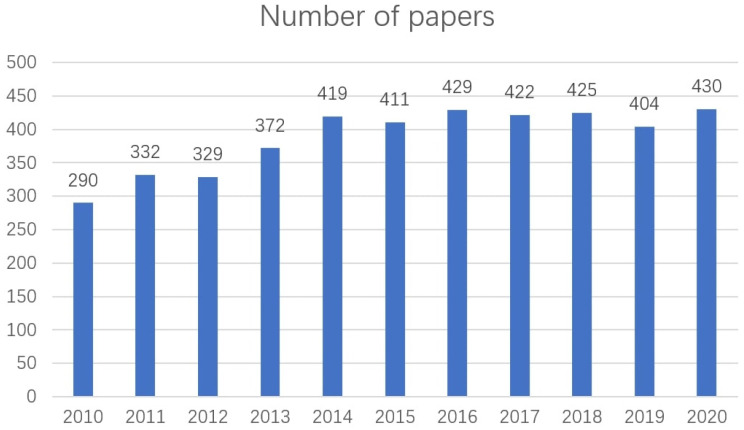
Number of papers about EBRT for rectal cancer area from 2010 to 2020.

**Figure 2:**
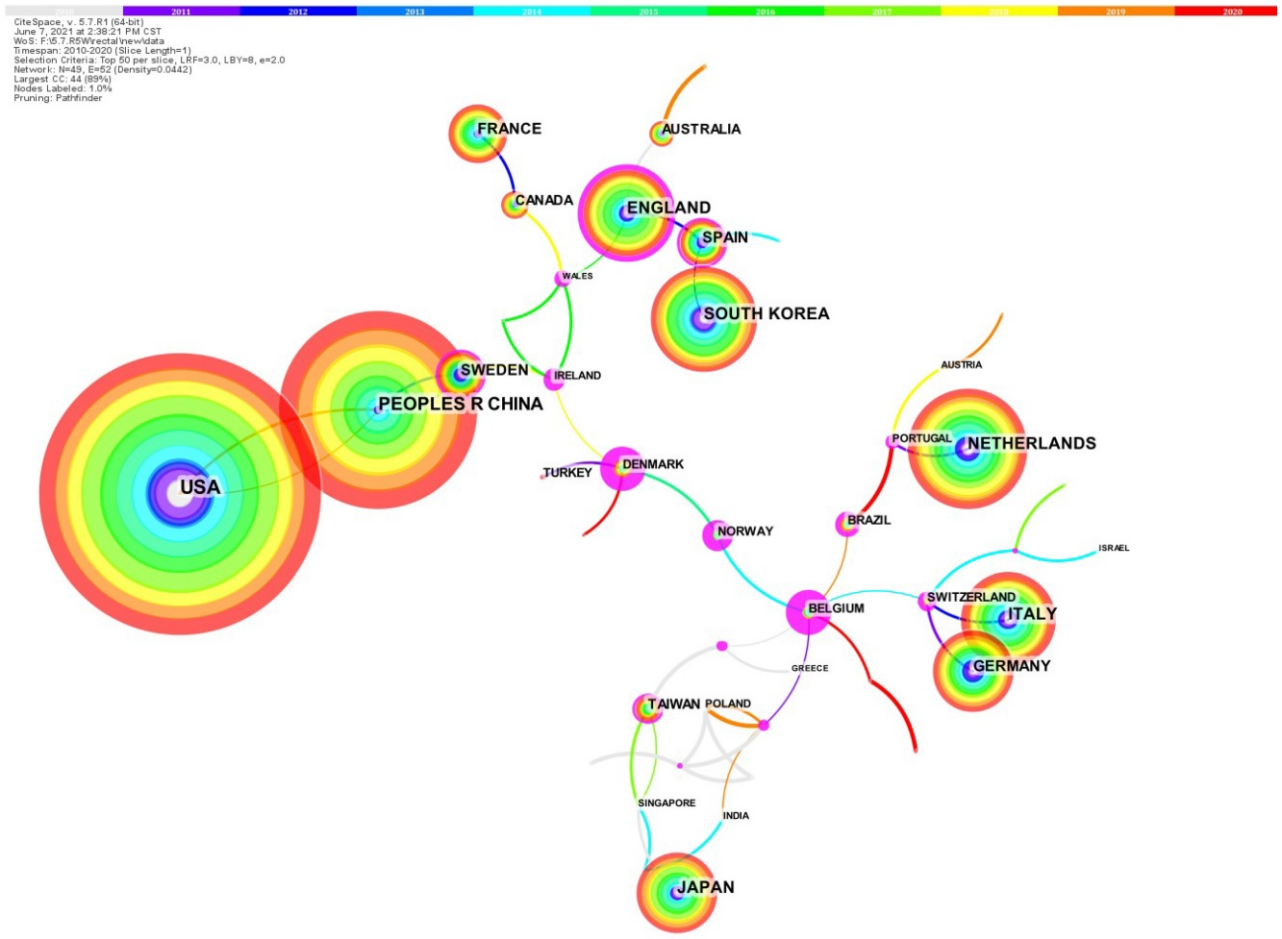
The country collaboration network.

WoSCC is one of the biggest databases global including SCI-EXPANDED, A&HCI, SSCI, BKCI-S, etc., which is containing peer-reviewed article published, and data from this database are commonly selected in bibliometric studies 6, 7. The time frame of this search was literature published between 2010 and 2020 (Figure 1). Document types were bounded to treatises and language was bounded to English (Figure 2) 5. The final screening yielded 4263 documents. Please see Supplementary Material S1 for the specific search strategy.

### Data analysis and visualization

The complete records and cited references of all 4263 papers were extracted in WoSCC, and the obtained results were exported as plain text and counted in Microsoft Office Excel 2019. Microsoft Office Excel 2019 was used to analyze the yearly trends in the number of publications in the literature. Knowledge mapping and bibliometric analysis of the exported plain text files were performed using CiteSpace (V. 5.7.R1) 8, 9.

## Results

### Papers characteristics on rectal cancer EBRT

A total of 4263 papers were searched (2010-2020) and analyzed 5. There is an increasing trend in the number of papers published each year from 2010 to 2020 (Figure 1) 5. These papers were written by 18,785 scholars who are from 89 countries/regions, 4618 institutes, and published in 531 different journals 5.

### National cooperative network of rectal cancer EBRT

All 4263 papers were published by a total of 89 regions/countries 5. The collaboration network among nations/regions that published papers from 2010 to 2020 (Figure 2) 5. Table S1 shows the information of the top 10 nations/regions, which are the highest number of published articles. Except for China, the top 10 countries with the highest productivity are all developed nations 5. These 10 nations published a total of 3803 articles, attributing to almost 90% of the total 5. Cross-country collaboration among the above 10 countries may help more articles to be published. Research institutions from the United States published 20.8% of the total number of articles (888), 1.5 times more than China (590). Centrality is also referred to as intermediate centrality. A higher centrality value of a node (country, institution, etc.) implies that it communicates and collaborates more with other nodes, or plays a more important role in collaborating with other nodes. The top three rankings for centrality are the England (0.35), Spain (0.15) and China (0.07). China is the only Asian country, suggesting that China may play a very important role in research collaboration and communication in rectal cancer.

### Establishing a collaborative network for EBRT in rectal cancer

**Figure 3:**
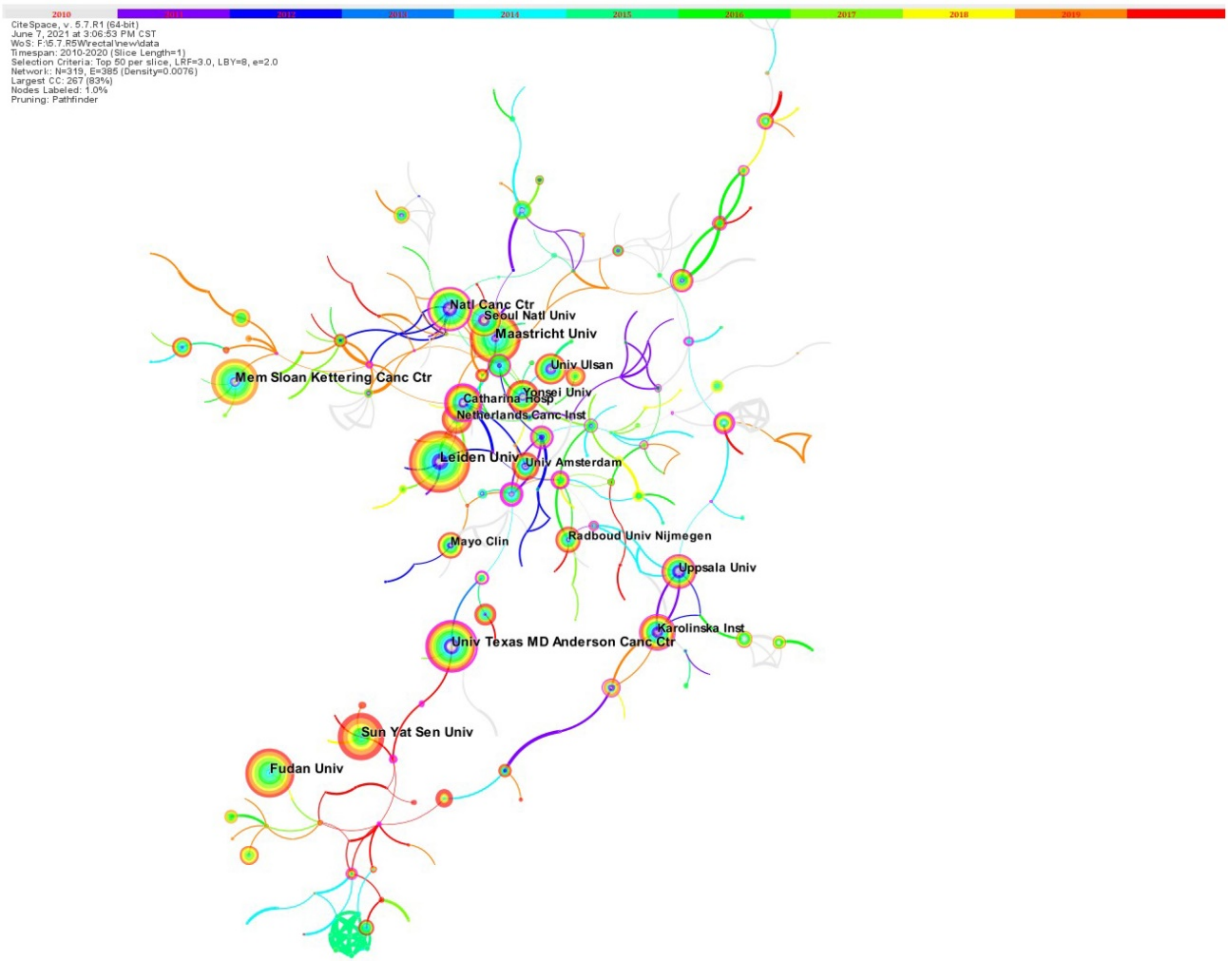
The institute collaboration network.

All 4263 papers were published by 4618 institutions. Figure 3 shows the network of collaboration among the institutions included in the literature. The density of the cooperation network is 0.0075, and overall the cooperation between institutions is very close. The shorter the distance between two circles, the more cooperation between the two institutions, and there is a lot of cooperation between Maastricht University and Seoul National University. Table S2 shows the top 10 high-output institutions, which published about 20.3% of the total number of papers. The top 10 institutions with the most published articles include 2 institutes in the Netherlands, 3 institutes in the USA and 3 in China. The larger the circle, the greater the number of posts. Leiden University is the institution with the highest number of published papers with 114 publications, followed by Maastricht University (96 publications), Fudan University (95 publications). The university Texas MD Anderson Cancer Center (0.32) has the highest central status, showing its very important role in the research field.

### Journal co citation network of rectal cancer EBRT

These 4263 articles were published on 531 journals, and the top 10 journals with the highest number of publications accounted for 29.6% of the total papers published. Among the top 10 most published journals (Table S3), *DISEASES of the COLON RECTUM* published the most papers (195 papers, 4.575%), followed by COLORECTAL DISEASE (183 papers, 4.293%).

**Figure 4:**
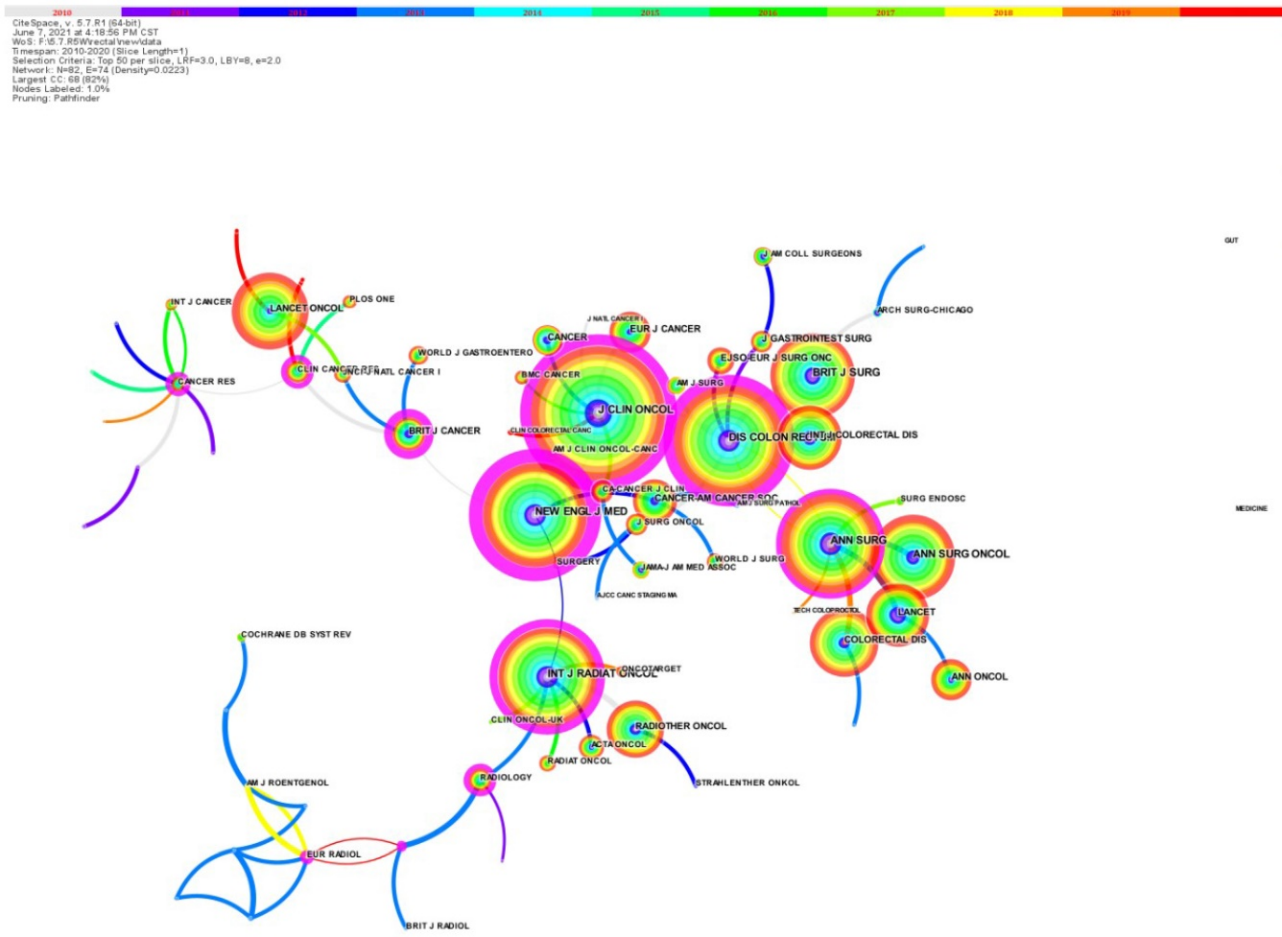
The journal co‐citation network.

The journal co-citation network is showed in Figure 4 (2010 to 2020) 5. Table S4 shows the top 10 most frequently cited journals. *JOURNAL of CLINICAL ONCOLOGY* is the most cited journal with 2775 citations, showing that this journal has had a profound impact on the conduct of clinical practice and other related research in this field, followed by *DISEASES of the COLON RECTUM* (542 articles) and *NEW ENGLAND JOURNAL of MEDICINE* (2196).

### Research hot spots

#### Co-citation network of papers on rectal cancer EBRT

**Figure 5:**
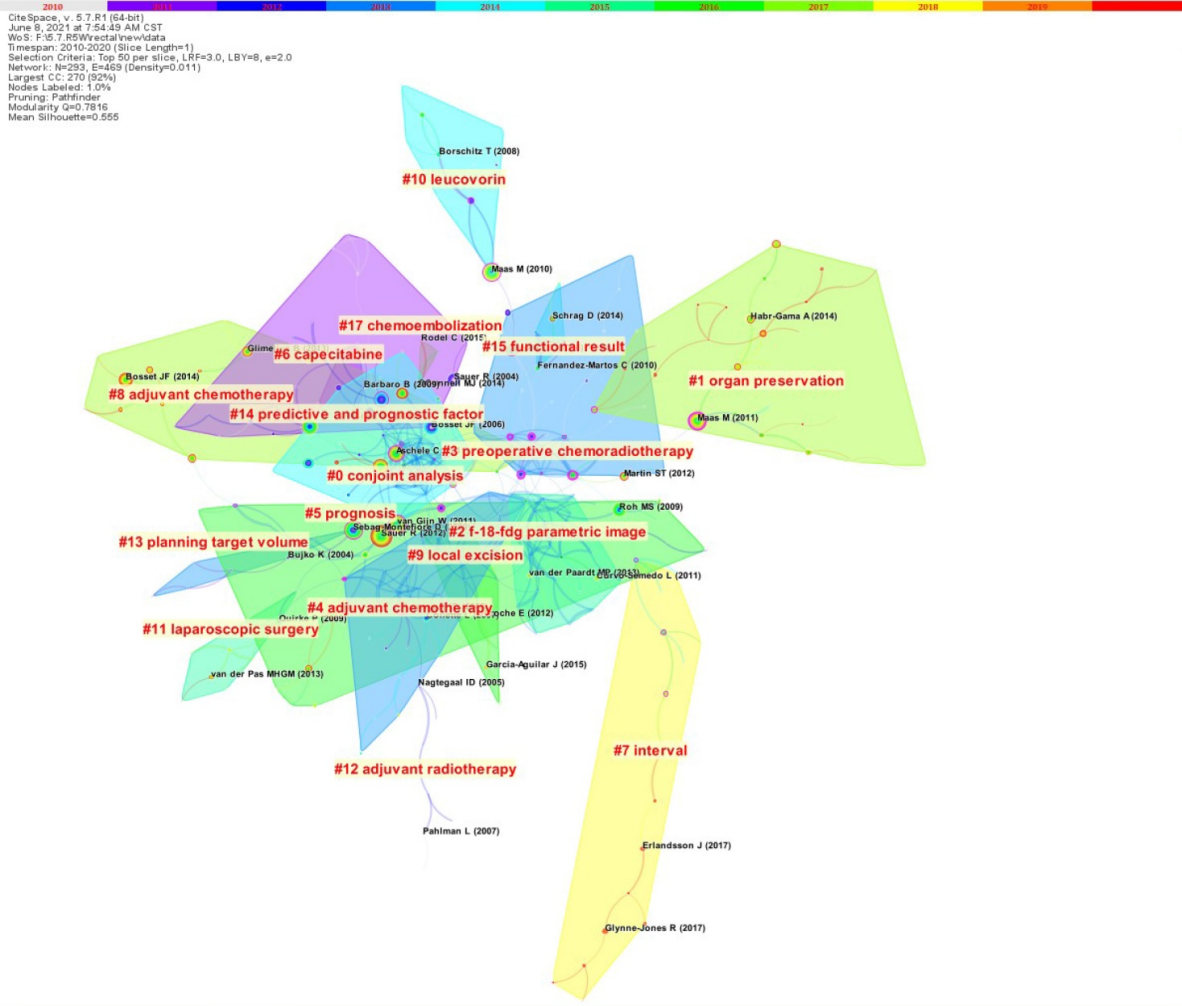
The paper co‐citation network.

The article co-citation network is showed (Figure 5) 5. The network has 293 nodes, 469 links and 17 main clusters, which have a modulus Q of 0.7816 and a mean profile of 0.555. The nodes and links show the references and citation relationships cited in the paper, respectively. The link colors directly reflect the time range, with cooler colors representing earlier years and warmer colors representing more recent years. Seventeen master clusters were got from CiteSpace (V. 5.7.R1), and these were labeled through using a log-likelihood ratio weighting algorithm and keyword terms (the algorithm calculates and determines the label for each type by using the core concept of each cluster given and a given specialty term) 5.

#### Citation data of the first four latest co citation clusters

Table S5 lists the top 4 most recent clusters: watch and wait, short-course radiotherapy, MRI, and neoadjuvant chemoradiotherapy. The paper focuses on the watch and wait strategy for patients with locally advanced rectal cancer after neoadjuvant therapy, i.e., with strict selection criteria and the latest imaging technology, watch and wait is feasible in complete clinical response (cCR) patients to achieve the goal of eliminating radical surgery and preserving the organ after neoadjuvant therapy 10-14. Five representative papers on “short-course radiotherapy” focus on the role of short-course radiotherapy in neoadjuvant regimens for rectal cancer, where the time interval between neoadjuvant treatment with short-course regimens and subsequent surgery can be moderately prolonged, and the addition of neoadjuvant chemotherapy during this period has a tendency to enhance the pathologic complete response (pCR) of patients (pathological complete remission) and does not increase complications 15-19. Five representative papers on “MRI” explored the “diagnostic and predictive role of MRI in rectal cancer”, by which the status of patients' lesions after neoadjuvant therapy can be better assessed and patients who achieve pCR can be more precisely screened for subsequent treatment choices, eliminating the need for standard surgical treatment if possible and reducing the potential side effects of patients during cancer treatment 20-24. The papers in the “neoadjuvant chemoradiotherapy” cluster are mainly about the improvement of neoadjuvant chemotherapy regimens and the addition of neoadjuvant post-adjuvant chemotherapy 25-29. Tables S6-S9 show the results separately 5.

### Reference burst in rectal cancer EBRT

Citation bursts are papers that have recently received significant citations and can reflect, to some extent, the research dynamics and hot spots in the field. From 2010 to 2020, a total of 103 references were marked as citation bursts (Table S10), highlighting the hot spots and trends in the field during this period. 27 references became the strongest bursts of citations that lasted until 2020, which reflected the latest hot spots in the field (Table S10) 10, 12-16, 18, 20, 25-42.

The more papers related to a topic then suggest the hotter the research in this field, after the analysis of the literature a total of 3 topics were obtained by screening, the rest of the topics related to a smaller number of literature (less than 4) were not focused on. The 1^st^ 22, 12^th^ 31, 23^rd^ 40 and 26^th^ 40 were mainly focused on the use of MRI in rectal cancer, 2^nd^ 14, 9^th^ 25, 10^th^ 26 and 15^th^ 33 were mainly about chemotherapy regimen improvement. The 11^th^ 27, 3^rd^ 43, 18^th^ 36, 20^th^ 18, 8^th^ 30 were related to the increase of adjuvant chemotherapy. Further, the 25^th^ 41 has the highest reference strength.

## Discussion

### General Data

The area of rectal cancer EBRT has progressed rapidly over the last few decades and the number of published papers is increasing 5. 9 of the 10 most fruitful nations are developed nations 5. Collaboration between countries is very strong, especially among Western countries. Among them, the research institutions from the England have the highest centrality, which may suggest that it has a clear benefit over other nations in a particular research direction, leading to a high level of cooperation with other nations 5. Japan, China and Korea are the only three Asian nations which make it into the top ten countries in terms of number of publications, but their collaboration intensity is less than that of Oceanian countries, North American, and European 5. In the future, it is hoped that these 3 countries will further deepen their cooperation and mutually promote the development of new research.

### Citation data

DISEASES OF THE COLON RECTUM is ranked first in the number of papers published in all 531 academic journals. The journal is ranked 2^nd^ in the number of citations, indicating the importance of the journal in this field. The top 10 authors in terms of the number of published papers each published at least 47 papers, and they are the 10 scholars who have achieved the most research results in this field.

### Co leading cluster hot spots

#### Watch and wait

Neoadjuvant radiotherapy can lead to downstaging of the tumor and some patients can even achieve clinical complete remission [cCR], but there is considerable controversy about what treatment regimen should be followed in patients who achieve cCR. Although not all patients with cCR will relapse will, the relapse possibility is still unsure, especially in patients with high or intermediate risk 5. It has been suggested that even if cCR is achieved, the patient should still undergo surgery.

The recent emergence of the watch and wait protocol offers a promising option for the design of next steps in treatment, and the results of Monique Maas' study suggest that with strict selection criteria, the latest imaging techniques, and regular follow-up, patients receiving the watch and wait protocol have prognostic OS and DFS as good as those who achieve pCR postoperatively, suggesting that the watch and wait strategy is feasible and the selection criteria and follow-up requirements in this study may provide a template for the design of subsequent randomized study protocols 10. Angelita Habr-Gama et al. reviewed that patients who achieved (cCR) after radiotherapy (CRT) and chose the watch and wait strategy, approximately 31% may experience local recurrence, but more than 90% of recurrences can be salvaged and did not affect overall patient survival, laterally supporting this choice 11. Ane L Appelt et al. concluded that high-dose radiotherapy and watchful waiting may be a safe alternative to abdominoperineal resection for patients with distal rectal cancer 12. The results of another study by Angelita Habr-Gama et al. showed that 50% of patients who achieved cCR after radiotherapy and did underwent surgery had satisfactory long-term outcomes, which, despite the small sample size, still further supports the watch and wait regimen 13.

#### Short-course radiotherapy

The improvement of radiotherapy regimens has been explored in recent years and related studies have been published one after another. A study designed by Johan Erlandsson et al. compared the difference in efficacy and side effects between immediate and delayed surgery after short-course radiotherapy 44. The results suggest that delayed surgery after short-course radiotherapy is an effective new regimen with significantly fewer postoperative complications while ensuring similar efficacy to the original regimen, and can be an alternative to the original short-course radiotherapy followed by immediate surgery 15, 44, which may be because the edema at the irradiated site has had sufficient time to subside. In the trial conducted by Per J Nilsson et al, a neoadjuvant regimen consisting of short-course radiotherapy with full-dose chemotherapy has advantages over the original neoadjuvant concurrent radiotherapy regimen, and more studies are needed in the future to further verify its ability to improve patient survival 18.

#### MRI

The current staging system is not refined enough for patient population delineation, and new tools are needed for more detailed delineation of rectal cancer patients to facilitate subsequent individualized treatment planning. MRI has shown unique advantages for assessing the status of lesions in rectal cancer patients, especially for cases that cannot be identified by other imaging techniques. MRI can better assess patients' circumferential margins, T-stage and lymph node status, etc., for more precise staging and selection of appropriate treatment strategies. Recently, studies have been reporting the role of MRI in determining the prognosis of rectal cancer. On the one hand, patients with potentially better prognosis are screened out before treatment and receive less neoadjuvant therapy, reducing the possible side effects of patients during treatment while saving medical resources. A study by Fiona G M Taylor et al. showed that MRI was able to screen out patients who only needed to receive surgery alone to achieve a good outcome 22. On the other hand, MRI evaluation of patients after neoadjuvant therapy better determines the outcome of treatment. A study by Doenja M J Lambregts et al. found that diffusion-weighted MRI (DWI) could better identify patients who reached ypT0 after neoadjuvant therapy 21.

#### Neoadjuvant chemoradiotherapy

The current neoadjuvant regimens have made great progress but are not yet satisfactory. Various attempts have been made in various research centers on how to further optimize the existing regimens as a next step. Michael J O'Connell et al. reported that the addition of oxaliplatin to capecitabine combined with preoperative radiotherapy did not further improve the efficacy but significantly increased the toxicity 26. However, data from Claus Rödel et al. showed that adding oxaliplatin to fluorouracil-based neoadjuvant radiotherapy and adjuvant chemotherapy significantly increased disease-free survival in rectal cancer patients (clinically staged cT3-4 or cN1-2) and could be a new treatment option for locally advanced rectal cancer 25, 45. Regarding the exploration of adding adjuvant chemotherapy after neoadjuvant radiotherapy, a multicenter, phase 2 trial conducted by Julio Garcia-Aguilar et al. showed that mFOLFOX6 administered after neoadjuvant radiotherapy may increase the rate of patients achieving pathologic complete response, and a phase 3 clinical trial is currently underway 27.

### Citation hot pots

#### Application of MRI in rectal cancer

MRI can provide more accurate information than conventional imaging techniques, thus helping clinicians to gain a more accurate understanding of the patient's current reality. According to data from a study by Fiona G M Taylor et al, the assessment of circumferential resection margin (CRM) status by high-resolution MRI correlated significantly with the risk of distant metastases and was superior to the AJCC TNM criteria for indicators such as LR, DFS, and OS 39. Data by Eisar Al-Sukhni et al similarly demonstrated this and suggested that, where available MRI should be taken for more accurate assessment of patients' CRM and T 42.

Moreover, with the continuous advancement of MRI technology, its role in various branches of medicine is becoming more important. But MRI can play those roles specifically in rectal cancer, different institutions have different ideas.

Uday B Patel et al. focused on the role of MRI for the evaluation of treatment outcome and the results of this team showed that MRI can provide a better assessment of the treatment outcome of neoadjuvant radiotherapy 31. It also identified ypT and CRM as important predictors, TRG and CRM as imaging markers for predicting survival outcomes, which can be referred to before proceeding with surgical treatment to decide whether additional treatment should be added 31.

MRI can also help in the prognosis of patients, and a study designed by Fiona G M Taylor et al. confirmed the ability of MRI to screen for patients who require only surgical treatment alone for a favorable outcome. The results point the way to some extent for further exploration of future studies 22.

#### Improvement of chemotherapy regimen

Chemotherapy plays an important role in the comprehensive treatment of rectal cancer, and its coordination mode with radiotherapy and surgery takes an important place in the survival rate of rectal cancer patients 46. However, new drugs are emerging with better efficacy and fewer side effects with the development of research techniques and continuous improvement of processes. So, it is receiving more attention how to form a more perfect integrated treatment plan about these new drugs combined with surgical radiotherapy and other therapies.

Data from Jean-Pierre Gérard et al. showed no significant difference in clinical efficacy with the addition of oxaliplatin in the existing neoadjuvant concurrent radiotherapy regimen of capecitabine combined with radiotherapy, and did not recommend this regimen for clinical use 14. Meanwhile, another study reported that the addition of oxaliplatin significantly increased toxicity 26. However, data and conclusions from other scholars are inconsistent. R Glynne-Jones et al. designed another study to evaluate the role of capecitabine and oxaliplatin in patients who had completed neoadjuvant therapy and surgery, during the adjuvant chemotherapy phase 33. However, due to the small number of patients, no valuable conclusions were obtained subsequent trials need to be redesigned for an in-depth exploration.

#### Increasing adjuvant chemotherapy

With the continuous exploration by scholars and clinicians physicians in various countries, the survival time of rectal cancer patients has been greatly enhanced by the existing combination of treatment options. Whether increasing the intensity of treatment (e.g., adjuvant chemotherapy) at the beginning of the existing treatment paradigm will further enhance the efficacy or even translate into survival benefit for patients has attracted increasing attention.

A phase 2 clinical study by Julio Garcia-Aguilar et al. unexpectedly found that the addition of adjuvant chemotherapy after radiotherapy and before surgery has the potential to convert patients to minimally invasive surgery for subsequent procedures with guaranteed efficacy; a related phase 3 clinical trial is underway 20. Also studies from Korea have shown- that preoperative radiotherapy and postoperative FOLFOX adjuvant chemotherapy regimens further improve disease-free survival than fluorouracil combined with folinic acid regimens 30. However, the long-term survival results reported in the EORTC 22921 study showed no improvement in OS or DFS with the addition of adjuvant chemotherapy after preoperative radiotherapy (combined with or without chemotherapy) 20. The PROCTOR-SCRIPT trial also failed to demonstrate a benefit of preoperative (chemoradiotherapy) and adjuvant chemotherapy after surgery on OS or DFS 47.

#### Other possible hot spots

Magnetic resonance imaging (MRI) is often used for evaluation during the treatment of rectal cancer due to its perfect soft tissue identification, and the creation and apply of MRI-LINAC has led to more accurate lesions and made adaptive radiotherapy for rectal cancer possible 5. Daily schedule adjustments may result in higher doses to the target area, reducing the range of off-target radiation on MRI-LINAC and further reducing the dose to organs at risk 5, 48. So, MRI linear gas pedals have also emerged as a potential research hotspot.

Total neoadjuvant therapy (TNT), which brings forward postoperative adjuvant chemotherapy for rectal cancer to the preoperative period, aims to improve patient compliance and long-term survival 49. TNT not only intervenes in micrometastases at an earlier stage; compared with postoperative radiotherapy, TNT also provides early symptom control with higher patient compliance and tolerance, thus ensure treatment intensity; for patients who achieve complete clinicopathological remission, they can choose to wait for observation and thus circumvent surgery 50. Subsequent long-term follow-up will verify whether it can contribute to improved survival 51. In addition, due to the high CR rate of patients receiving TNT regimens and its organ preservation features, it may help to enhance the non-surgical component of comprehensive oncology treatment regimens in the future 51, 52. Therefore, TNT has become a highly promising research direction.

### Advantages and disadvantages

This bibliometric analysis of rectal cancer EBRT helps researchers to understand the hot spots and future research trends in the field. By searching and filtering the literature from WoSCC and conducting the corresponding analysis, a more objective, comprehensive and visualized understanding of the field can be provided, which can provide a wealth of information about the field and even help researchers to get a quick introduction to the field in a short period of time.

However, this study also has some drawbacks. First, only relevant literature data were collected from WoSCC, and other databases (such as Google Scholar) were not searched and filtered accordingly 5. Next, most of the articles were published in English, and other language articles were not contained 5. Then, because some papers published in recent years are close to the date of search, the corresponding citation counts are not high enough, so the results of quantitative analysis and the actual position of the relevant research in the field may be biased. Fourth, there may be some missing items in the plain text downloaded from WoSCC 5. Finally, although this study included the vast majority of the literature, the analysis did not include literature published after 2021. Nevertheless, this study covers the vast majority of the literature published since 2010, and the small number of papers not included may not significantly affect the overall trend of this study.

## Conclusion

In summary, “watch and wait, short-course radiotherapy, MRI and neoadjuvant chemoradiotherapy”, “MRI in rectal cancer”, and “chemotherapy regimen improvement” are probably the hottest areas of research at the moment. For novice researchers entering the field, they should focus on these four most cited clusters and three hot pots. This study has important implications for the treatment of rectal cancer with EBRT, especially useful for clinical decision making by health care providers and for the management of patients with rectal cancer 5.

## Supplementary Material

Supplementary appendices and tables.

## Author Contributions

QB and ZM conducted the search strategy, analyzed the data, wrote and revised the manuscript. SF and FZ critically revised the manuscript and provided the final approval of the manuscript. All authors read and approved the final manuscript.
