# Supplementary material for: Detecting the Research Trends and Hot Spots in External Irradiation Therapy for Rectal Cancer
Source: J Cancer. 2022 Apr 4;13(7):2179–88. doi: 10.7150/jca.69669 (PMC9066221; doi:10.7150/jca.69669)
Supplement: Supplementary file 1 — Supplementary appendices and tables. [file jcav13p2179s1.pdf]

Appendix S1

Search strategy (June 7th, 2021):

Select a database: Web of Science Core Collection

Document types: Article

Language: English

Duration: 2010.1-2020.12

Citation Indexes: The Science Citation Index Expanded (SCI-EXPANDED),

the Social Sciences Citation Index (SSCI),

the Conference Proceedings Citation Index-Science (CPCI-S),

the Conference Proceedings Citation Index-Social Science & Humanities (CPCI-SSH), the Book Citation Index-Science (BKCI-S),

the Index Chemicus (IC)

Results: 4263

Boolean operation rules:

((TS=(("rectum cancer") OR ("Rectal Cancers") OR ("Neoplasm, Rectal") OR ("Rectal Neoplasm") OR ("Rectum Neoplasms") OR ("Neoplasm, Rectum") OR ("Rectum Neoplasm") OR ("Rectal Tumors") OR ("Rectal Tumor") OR ("Tumor, Rectal") OR ("Neoplasms, Rectal") OR ("Cancer of Rectum") OR ("Rectum Cancers") OR ("Rectum Cancers") OR ("Rectal Cancer") OR ("Rectum Cancer") OR ("Cancer, Rectum") OR ("Cancer of the Rectum") OR ("Cancer, Rectal")))AND ((TS=(("radiation oncology") OR ("radiation therapy") OR (radiotherapy) OR ("conformal radiation therapy") OR ("conformal radiotherapy") OR ("external beam radiation therapy") OR ("external beam radiotherapy") OR (EBRT) OR ("intensity-modulated radiation therapy") OR ("intensity modulated radiation therapy") OR ("intensity-modulated radiotherapy") OR ("intensity modulated radiotherapy") OR (IMRT) OR ("stereotacticbody radiotherapy") OR ("neo-adjuvant chemoradiation") OR ("neoadjuvant chemoradiation") OR ("adjuvant chemoradiation") OR ("stereotacticbody radiation therapy") OR (SBRT) OR ("stereotactic radiosurgery") OR ("stereotactic radiotherapy") OR ("stereotactic radiation therapy") OR ("Image Guided Radiotherapy") OR ("Image Guided Radiation therapy") OR (IGRT) OR ("image guided adaptive radiotherapy") OR ("image guided adaptive radiation therapy") OR ("adaptive radiation therapy") OR ("adaptive radiotherapy") OR (IGART) OR (VMAT) OR ("Volumetric Intensity Modulated Arc Therapy") OR ("proton radiotherapy") OR ("proton radiation therapy"))) NOT (TS=(("Radioisotope Brachytherapy") OR ("Curietherapy") OR ("Interstitial Radiotherapy") OR ("Implant Radiotherapy") OR ("high dose rate") OR (HDR) OR ("low dose rate") OR (LDR) OR ("pulsed dose rate") OR (PDR) OR ("medium dose rate") OR (MDR) OR (brachytherapy)))))

Appendix S2

Supplementary Tables:

Table S 1. Top 10 countries based on count.

Table S 2. Top 10 institutes based on count.

Table S 3. Top 10 most productive journals.

Table S 4. Top 10 journals with most co-citation count.

Table S 5. Details of the 17 clusters.

Table S 6. The representative papers of Cluster 1.

Table S 7. The representative papers of Cluster 7.

Table S 8. The representative papers of Cluster 8.

Table S 9. The representative papers of Cluster 17.

Table S 10. Top103 References with the Strongest Citation Bursts.

Table S 1. Top 10 countries based on count.

Rank	Count	Centrality	Country
1	888	0	USA
2	590	0.07	PEOPLES R CHINA
3	387	0	NETHERLANDS
4	353	0	SOUTH KOREA
5	339	0	ITALY
6	294	0	JAPAN
7	292	0.35	ENGLAND
8	268	0	GERMANY
9	213	0	FRANCE
10	179	0.15	SPAIN

Table S 2. Top 10 institutes based on count.

Rank	Count	Centrality	Institutes
1	114	0.06	LEIDEN UNIVERSITY
2	96	0.09	MAASTRICHT UNIVERSITY
3	95	0.00	FUDAN UNIVERSITY
4	94	0.03	MEMORIAL SLOAN-KETTERING CANCER CENTER
5	92	0.32	UNIVERSITY TEXAS MD ANDERSON CANCER CENTER
6	86	0.25	NATIONAL CANCER CENTER
7	83	0.03	SUN YAT-SEN UNIVERSITY
8	71	0.28	CATHARINA HOSPITAL
9	69	0.11	KAROLINSKA INSTITUTION
10	69	0.03	YONSEI UNIVERSITY

Table S 3. Top 10 most productive journals

Rank	Journals	Count	% of 4,263	IF（2020）
1	DISEASES OF THE COLON RECTUM	195	4.575	3.99
2	COLORECTAL DISEASE	183	4.293	2.765
3	INTERNATIONAL JOURNAL OF RADIATION ONCOLOGY BIOLOGY	156	3.660	5.856

	PHYSICS			
4	INTERNATIONAL JOURNAL OF COLORECTAL DISEASE	132	3.097	2.108
5	RADIOTHERAPY AND ONCOLOGY	124	2.909	4.855
6	ANNALS OF SURGICAL ONCOLOGY	123	2.886	4.0608
7	EJSO	99	2.323	3.956
8	RADIATION ONCOLOGY	99	2.323	2.817
9	BMC CANCER	81	1.901	3.15
10	ONCOTARGET	69	1.619	-

Table S 4. Top 10 journals with most co-citation count.

Rank	Count	Centrality	Cited Journals	IF
1	2775	0.77	JOURNAL OF CLINICAL ONCOLOGY	32.9557
2	2277	0.65	DISEASES OF THE COLON RECTUM	3.9909
3	2196	0.95	NEW ENGLAND JOURNAL OF MEDICINE	
4	2140	0.6	INTERNATIONAL JOURNAL OF RADIATION	33.7515
5	2110	0.27	ANNALS OF SURGERY	10.1296
6	1827	0	ANNALS OF SURGICAL ONCOLOGY	4.0608
7	1825	0.08	BRITISH JOURNAL OF SURGERY	74.6982
8	1665	0.04	LANCET ONCOLOGY	5.8593
9	1439	0.04	LANCET	5.6759
10	1423	0.04	COLORECTAL DISEASE	2.7688

Table S 5. Details of the 17 clusters. （cluster summary of clusters）

Cluster ID	Size	Silhouette	Mean（Year）	Label (LLR)(4-5 个)
0	34	0.947	2009	conjoint analysis (9.31, 0.005); late effects (9.31, 0.005); randomized trials (6.28, 0.05); delineation atlas (4.65, 0.05)
1	29	1	2015	organ preservation (13.29, 0.001); radiomics (13.29, 0.001); watch and wait (13.12, 0.001); magnetic resonance imaging (10.11, 0.005)

2	25	0.979	2008	f-18-fdg parametric image (6.79, 0.01); dual-time f-18-fdg pet (6.79, 0.01); gross target volume (6.79, 0.01); diffusion-weighted mri (6.79, 0.01);
3	25	0.909	2006	preoperative chemoradiotherapy (6.92, 0.01); capecitabine (5.59, 0.05); tumour regression (5.22, 0.05); zap70 (5.22, 0.05)
4	24	0.875	2008	adjuvant chemotherapy (11.36, 0.001); adenocarcinoma (6.37, 0.05); delphi method (5.84, 0.05); near-complete (5.84, 0.05)
5	22	0.921	2010	prognosis (9.98, 0.005); elderly (5.14, 0.05); immunotherapy (5.14, 0.05); anal canal (4.36, 0.05)
6	21	1	2006	capecitabine (10.88, 0.001); cetuximab (5.76, 0.05); anal cancer (5.33, 0.05); radiation (5.04, 0.05); sphincter preservation (4.74, 0.05)
7	16	0.984	2014	interval (21.98, 1.0E-4); short-course radiotherapy (13.16, 0.001); texture analysis (13.16, 0.001); mucinous adenocarcinoma (8.76, 0.005)
8	15	0.962	2013	adjuvant chemotherapy (10.91, 0.001); circumferential resection margin (9.3, 0.005); MRI (5.86, 0.05); pathologic complete response (6.18, 0.05)
9	13	0.911	2010	local excision (20.85, 1.0E-4); watch and wait (10.43, 0.005); rectum-preserving approach (7.8, 0.01); lymphoscintigraphy (7.8, 0.01); tem surgery (7.8, 0.01)
10	11	1	2006	leucovorin (7.45, 0.01); post-treatment rectal cancer (6.4, 0.05); minimally invasive approach (6.4, 0.05); resection margins (6.4, 0.05)
11	9	1	2011	laparoscopic surgery (18.89, 1.0E-4); laparoscopy (15.94, 1.0E-4); oncological outcomes (12.57, 0.001); robotic surgical procedures (12.57, 0.001)
12	7	0.979	2005	adjuvant radiotherapy (10.63, 0.005); disparities (8.6, 0.005); helical tomotherapy (8.6, 0.005); simultaneous integrated boost (8.6, 0.005)
13	6	1	2006	planning target volume (7.28, 0.01); tktl1 (7.28, 0.01); hypoxia (7.28, 0.01); rectal motion (7.28, 0.01)
14	5	0.991	2005	predictive and prognostic factor (8.37, 0.005); bispecific antibody (8.37, 0.005); histologic regression (8.37, 0.005); response evaluation (8.37, 0.005)
15	5	0.986	2010	functional result (7.55, 0.01); second mitochondria-derived activator of caspase vascular endothelial growth factor (7.55, 0.01); colorectal adenocarcinoma (7.55, 0.01); helical (7.55, 0.01)
17	3	0.978	2014	chemoembolization (7.11, 0.01); clinical study (7.11, 0.01); neoadjuvant chemoradiotherapy (6.07, 0.05); locally-advanced rectal cancer (4.4, 0.05)

Table S 6. The representative papers of Cluster 1.

Serial Numbe r	Title

1	Wait-and-see policy for clinical complete responders after chemoradiation for rectal cancer
2	Local recurrence after complete clinical response and watch and wait in rectal cancer after neoadjuvant chemoradiation: impact of salvage therapy on local disease control
3	High-dose chemoradiotherapy and watchful waiting for distal rectal cancer: a prospective observational study
4	Watch and wait approach following extended neoadjuvant chemoradiation for distal rectal cancer: are we getting closer to anal cancer management?
5	Clinical outcome of the ACCORD 12/0405 PRODIGE 2 randomized trial in rectal cancer

Table S 7. The representative papers of Cluster 7.

Serial Number	Title
1	Optimal fractionation of preoperative radiotherapy and timing to surgery for rectal cancer (Stockholm III): a multicentre, randomised, non-blinded, phase 3, non-inferiority trial
2	Effect of Interval (7 or 11 weeks) Between Neoadjuvant Radiochemotherapy and Surgery on Complete Pathologic Response in Rectal Cancer: A Multicenter, Randomized, Controlled Trial (GRECCAR-6)
3	Optimal time interval between neoadjuvant chemoradiotherapy and surgery for rectal cancer
4	Short-course radiotherapy followed by neo-adjuvant chemotherapy in locally advanced rectal cancer--the RAPIDO trial
5	Optimal timing of surgery after chemoradiation for advanced rectal cancer: preliminary results of a multicenter, nonrandomized phase II prospective trial

Table S 8. The representative papers of Cluster 8.

Serial Number	Title
1	Fluorouracil-based adjuvant chemotherapy after preoperative chemoradiotherapy in rectal cancer: long-term results of the EORTC 22921 randomised study
2	Tumour ADC measurements in rectal cancer: effect of ROI methods on ADC values and interobserver variability
3	Preoperative high-resolution magnetic resonance imaging can identify good prognosis stage I, II, and III rectal cancer best managed by surgery alone: a prospective, multicenter, European study
4	Adjuvant chemotherapy after preoperative (chemo)radiotherapy and surgery for patients with rectal cancer: a systematic review and meta-analysis of individual patient data
5	Diffusion-weighted MRI for selection of complete responders after chemoradiation for locally advanced rectal cancer: a multicenter study

Table S 9. The representative papers of Cluster 17.

Serial Number	Title

1	Oxaliplatin added to fluorouracil-based preoperative chemoradiotherapy and postoperative chemotherapy of locally advanced rectal cancer (the German CAO/ARO/AIO-04 study): final results of the multicentre, open-label, randomised, phase 3 trial
2	Capecitabine and oxaliplatin in the preoperative multimodality treatment of rectal cancer: surgical end points from National Surgical Adjuvant Breast and Bowel Project trial R-04
3	Effect of adding mFOLFOX6 after neoadjuvant chemoradiation in locally advanced rectal cancer: a multicentre, phase 2 trial

Table S 10. Top103 References with the Strongest Citation Bursts.

Rank	References	Year	Strength	Begin	End	2010 - 2020
1	Birgisson H, 2005, J CLIN ONCOL, V23, P8697, DOI	2005	9.7303	**2010**	2011	
2	Vecchio FM, 2005, INT J RADIAT ONCOL, V62, P752, DOI	2005	16.0515	**2010**	2013	
3	Brown G, 2006, BRIT MED J, V333, P779, DOI	2006	15.4385	**2010**	2014	
4	Nagtegaal ID, 2008, J CLIN ONCOL, V26, P303, DOI	2008	9.0443	**2010**	2014	
5	Hurwitz H, 2004, NEW ENGL J MED, V350, P2335, DOI	2004	6.8796	**2010**	2012	
6	Peeters KCMJ, 2005, J CLIN ONCOL, V23, P6199, DOI	2005	21.0731	**2010**	2012	
7	Habr-Gama A, 2004, ANN SURG, V240, P711, DOI	2004	23.3402	**2010**	2012	
8	Bujko K, 2006, BRIT J SURG, V93, P1215, DOI	2006	29.0577	**2010**	2014	
9	Gunderson LL, 2004, J CLIN ONCOL, V22, P1785, DOI	2004	6.4487	**2010**	2012	
10	Pahlman L, 2007, BRIT J SURG, V94, P1285, DOI	2007	8.6042	**2010**	2012	
11	Nagtegaal ID, 2005, J CLIN ONCOL, V23, P9257, DOI	2005	7.3106	**2010**	2012	
12	Mohiuddin M, 2006, J CLIN ONCOL, V24, P650, DOI	2006	15.5216	**2010**	2012	
13	Gerard JP, 2006, J CLIN ONCOL, V24, P4620, DOI	2006	62.5156	**2010**	2014	
14	Sauer R, 2004, NEW ENGL J MED, V351, P1731, DOI	2004	157.2532	**2010**	2012	
15	Marijnen CAM, 2005, J CLIN ONCOL, V23, P1847, DOI	2005	15.2448	**2010**	2013	
16	Stipa F, 2006, ANN SURG ONCOL, V13, P1047, DOI	2006	10.6361	**2010**	2011	
17	Bosset JF, 2005, J CLIN ONCOL, V23, P5620,	200	18.5576	**2010**	201	

	DOI	5			2
18	Folkesson J, 2005, J CLIN ONCOL, V23, P5644, DOI	2005	18.8787	**2010**	2013
19	Bujko K, 2004, RADIOTHER ONCOL, V72, P15, DOI	2004	13.0624	**2010**	2011
20	Bosset JF, 2006, NEW ENGL J MED, V355, P1114, DOI	2006	88.9866	**2010**	2014
21	Rodel C, 2007, J CLIN ONCOL, V25, P110, DOI	2007	13.3567	**2010**	2012
22	Rodel C, 2005, J CLIN ONCOL, V23, P8688, DOI	2005	40.5083	**2010**	2012
23	Garcia-Aguilar J, 2003, DIS COLON RECTUM, V46, P298, DOI	2003	13.548	**2010**	2011
24	Peeters KCMJ, 2007, ANN SURG, V246, P693, DOI	2007	29.1225	**2010**	2015
25	Guillem JG, 2008, J CLIN ONCOL, V26, P368, DOI	2008	12.5768	**2010**	2011
26	Collette L, 2007, J CLIN ONCOL, V25, P4379, DOI	2007	14.1328	**2010**	2015
27	Capirci C, 2008, INT J RADIAT ONCOL, V72, P99, DOI	2008	8.936	**2011**	2012
28	Das P, 2007, CANCER-AM CANCER SOC, V109, P1750, DOI	2007	9.2045	**2011**	2012
29	Borschitz T, 2008, ANN SURG ONCOL, V15, P712, DOI	2008	11.1089	**2011**	2016
30	Valentini V, 2009, RADIOTHER ONCOL, V92, P148, DOI	2009	14.4947	**2011**	2014
31	Barbaro B, 2009, RADIOLOGY, V250, P730, DOI	2009	8.413	**2011**	2012
32	Bipat S, 2004, RADIOLOGY, V232, P773, DOI	2004	12.6336	**2011**	2012
33	Willett CG, 2009, J CLIN ONCOL, V27, P3020, DOI	2009	9.4673	**2011**	2012
34	Habr-Gama A, 2006, J GASTROINTEST SURG, V10, P1319, DOI	2006	6.9395	**2011**	2013
35	Tulchinsky H, 2008, ANN SURG ONCOL, V15, P2661, DOI	2008	9.5191	**2011**	2014
36	Chan I, 2006, J CLIN ONCOL, V24, P668, DOI	2006	9.9451	**2012**	2014
37	Elferink MAG, 2010, EUR J CANCER, V46, P1421, DOI	2010	4.276	**2012**	2013
38	Radu C, 2008, RADIOTHER ONCOL, V87, P343, DOI	2008	4.276	**2012**	2013
39	Birgisson H, 2007, ACTA ONCOL, V46, P504,	200	3.7671	**2012**	201

	DOI	7			3
40	Fernandez-Martos C, 2010, J CLIN ONCOL, V28, P859, DOI	2010	10.5655	**2012**	2015
41	Roels S, 2006, INT J RADIAT ONCOL, V65, P1129, DOI	2006	4.276	**2012**	2013
42	Chua YJ, 2010, LANCET ONCOL, V11, P241, DOI	2010	12.4182	**2012**	2014
43	Hofheinz RD, 2012, LANCET ONCOL, V13, P0, DOI	2012	11.2815	**2013**	2018
44	Glynne-Jones R, 2012, BRIT J SURG, V99, P897, DOI	2012	5.1099	**2013**	2014
45	Kim SH, 2009, RADIOLOGY, V253, P116, DOI	2009	7.431	**2013**	2014
46	Edge SB, 2010, AJCC CANC STAGING MA, V0, P0	2010	11.4143	**2013**	2015
47	Kalady MF, 2009, ANN SURG, V250, P582, DOI	2009	16.0927	**2014**	2017
48	Sebag-Montefiore D, 2009, LANCET, V373, P811, DOI	2009	9.8824	**2014**	2015
49	Siegel R, 2012, CA-CANCER J CLIN, V62, P10, DOI	2012	12.6098	**2014**	2015
50	Lezoche E, 2012, BRIT J SURG, V99, P1211, DOI	2012	14.2521	**2014**	2017
51	Quirke P, 2009, LANCET, V373, P821, DOI	2009	4.9239	**2014**	2015
52	Roh MS, 2009, J CLIN ONCOL, V27, P5124, DOI	2009	12.5993	**2014**	2017
53	Petersen SH, 2012, COCHRANE DB SYST REV, V0, P0, DOI	2012	11.1502	**2014**	2015
54	Taylor FGM, 2011, ANN SURG, V253, P711, DOI	2011	5.1457	**2014**	2020
55	Gerard JP, 2010, J CLIN ONCOL, V28, P1638, DOI	2010	2.7251	**2014**	2015
56	Siegel R, 2013, CA-CANCER J CLIN, V63, P11, DOI	2013	11.6366	**2014**	2015
57	Garcia-Aguilar J, 2012, ANN SURG ONCOL, V19, P384, DOI	2012	14.8617	**2014**	2017
58	Gerard JP, 2012, J CLIN ONCOL, V30, P4558, DOI	2012	15.6409	**2015**	2020
59	Maas M, 2010, LANCET ONCOL, V11, P835, DOI	2010	29.3195	**2015**	2018
60	Smith JD, 2012, ANN SURG, V256, P965, DOI	2012	18.4501	**2015**	2017
61	van Gijn W, 2011, LANCET ONCOL, V12,	201	17.3771	**2015**	201

	P575, DOI	1			7
62	Braendengen M, 2008, J CLIN ONCOL, V26, P3687, DOI	2008	4.8069	**2015**	2016
63	Bosset JF, 2014, LANCET ONCOL, V15, P184, DOI	2014	16.0421	**2015**	2020
64	Habr-Gama A, 2014, INT J RADIAT ONCOL, V88, P822, DOI	2014	11.3649	**2015**	2018
65	Aschele C, 2011, J CLIN ONCOL, V29, P2773, DOI	2011	10.9674	**2015**	2017
66	Rodel C, 2012, LANCET ONCOL, V13, P679, DOI	2012	9.4655	**2015**	2016
67	Dewdney A, 2012, J CLIN ONCOL, V30, P1620, DOI	2012	15.1142	**2015**	2017
68	Pettersson D, 2010, BRIT J SURG, V97, P580, DOI	2010	11.6998	**2015**	2016
69	Martin ST, 2012, BRIT J SURG, V99, P918, DOI	2012	11.9051	**2015**	2020
70	Maas M, 2011, J CLIN ONCOL, V29, P4633, DOI	2011	14.0656	**2015**	2020
71	Habr-Gama A, 2010, DIS COLON RECTUM, V53, P1692, DOI	2010	9.5647	**2016**	2017
72	Ngan SY, 2012, J CLIN ONCOL, V30, P3827, DOI	2012	6.6871	**2016**	2017
73	Fokas E, 2014, J CLIN ONCOL, V32, P1554, DOI	2014	10.6203	**2016**	2020
74	Habr-Gama A, 2013, DIS COLON RECTUM, V56, P1109, DOI	2013	16.7323	**2016**	2020
75	Glimelius B, 2013, ANN ONCOL, V24, P81, DOI	2013	17.1507	**2016**	2018
76	Curvo-Semedo L, 2011, RADIOLOGY, V260, P734, DOI	2011	16.1945	**2016**	2018
77	Hong YS, 2014, LANCET ONCOL, V15, P1245, DOI	2014	11.5852	**2016**	2020
78	Rodel C, 2015, LANCET ONCOL, V16, P979, DOI	2015	30.3378	**2016**	2020
79	Sloothaak DAM, 2013, BRIT J SURG, V100, P933, DOI	2013	15.0585	**2016**	2018
80	OConnell MJ, 2014, J CLIN ONCOL, V32, P1927, DOI	2014	13.9201	**2016**	2020
81	Garcia-Aguilar J, 2015, LANCET ONCOL, V16, P957, DOI	2015	17.3366	**2016**	2020
82	Lambregts DMJ, 2011, ANN SURG ONCOL, V18, P2224, DOI	2011	11.0557	**2016**	2017
83	Patel UB, 2011, J CLIN ONCOL, V29, P3753,	201	11.4741	**2016**	202

	DOI	1			0
84	van der Paardt MP, 2013, RADIOLOGY, V269, P101, DOI	2013	15.5572	**2016**	2018
85	Sauer R, 2012, J CLIN ONCOL, V30, P1926, DOI	2012	20.4587	**2016**	2020
86	Appelt AL, 2015, LANCET ONCOL, V16, P919, DOI	2015	16.7323	**2016**	2020
87	Edge SB, 2010, ANN SURG ONCOL, V17, P1471, DOI	2010	18.0899	**2016**	2018
88	van der Pas MHGM, 2013, LANCET ONCOL, V14, P210, DOI	2013	6.2169	**2017**	2018
89	Glynne-Jones R, 2014, ANN ONCOL, V25, P1356, DOI	2014	14.3344	**2017**	2020
90	Garcia-Aguilar J, 2015, LANCET ONCOL, V16, P1537, DOI	2015	16.6274	**2017**	2020
91	Stevenson ARL, 2015, JAMA-J AM MED ASSOC, V314, P1356, DOI	2015	10.7476	**2017**	2018
92	Renehan AG, 2016, LANCET ONCOL, V17, P174, DOI	2016	25.0436	**2017**	2020
93	Schmoll HJ, 2012, ANN ONCOL, V23, P2479, DOI	2012	6.8905	**2017**	2018
94	Breugom AJ, 2015, ANN ONCOL, V26, P696, DOI	2015	15.9031	**2017**	2020
95	Lefevre JH, 2016, J CLIN ONCOL, V34, P3773, DOI	2016	19.9409	**2018**	2020
96	Nilsson PJ, 2013, BMC CANCER, V13, P0, DOI	2013	15.2508	**2018**	2020
97	Deng YH, 2016, J CLIN ONCOL, V34, P3300, DOI	2016	22.933	**2018**	2020
98	Schrag D, 2014, J CLIN ONCOL, V32, P513, DOI	2014	8.5532	**2018**	2020
99	Taylor FGM, 2014, J CLIN ONCOL, V32, P34, DOI	2014	18.6604	**2018**	2020
100	Emmertsen KJ, 2012, ANN SURG, V255, P922, DOI	2012	22.5052	**2018**	2020
101	Glynne-Jones R, 2017, ANN ONCOL, V28, P22	2017	41.8728	**2018**	2020
102	Al-Sukhni E, 2012, ANN SURG ONCOL, V19, P2212, DOI	2012	7.4453	**2018**	2020
103	Erlandsson J, 2017, LANCET ONCOL, V18, P336, DOI	2017	24.217	**2018**	2020
